# AoP-LSE: Antioxidant Proteins Classification Using Deep Latent Space Encoding of Sequence Features

**DOI:** 10.3390/cimb43030105

**Published:** 2021-10-09

**Authors:** Muhammad Usman, Shujaat Khan, Seongyong Park, Jeong-A Lee

**Affiliations:** 1 Department of Computer Engineering, Chosun University, Gwangju 61452, Korea; usman@chosun.kr; 2 Department of Bio and Brain Engineering, KAIST, Daejeon 34141, Korea; shujaat@kaist.ac.kr (S.K.); sypark0215@kaist.ac.kr (S.P.)

**Keywords:** antioxidation, deep auto-encoder, composition of k-spaced amino acid pair (CKSAAP), latent space learning, neural network, classification

## Abstract

It is of utmost importance to develop a computational method for accurate prediction of antioxidants, as they play a vital role in the prevention of several diseases caused by oxidative stress. In this correspondence, we present an effective computational methodology based on the notion of deep latent space encoding. A deep neural network classifier fused with an auto-encoder learns class labels in a pruned latent space. This strategy has eliminated the need to separately develop classifier and the feature selection model, allowing the standalone model to effectively harness discriminating feature space and perform improved predictions. A thorough analytical study has been presented alongwith the PCA/tSNE visualization and PCA-GCNR scores to show the discriminating power of the proposed method. The proposed method showed a high MCC value of 0.43 and a balanced accuracy of 76.2%, which is superior to the existing models. The model has been evaluated on an independent dataset during which it outperformed the contemporary methods by correctly identifying the novel proteins with an accuracy of 95%.

## 1. Introduction

The oxidative damage due to free radicals is prevented by the antioxidants which are naturally produced in the human body [1]. Small antioxidant molecules, scavenges the free radicals by neutralizing them, where as the large antioxidant molecules prevents the potential damage by absorbing the free radicals [2]. However, on some occasions, the naturally produced antioxidants are outnumbered by the free radicals, and their procurement from the external sources including vegetables and fruits become indispensable [3,4]. The macro-molecules (proteins), that offer these nutrients must be effectively identified, since it would contribute towards the development of novel methods to prevent the diseases caused by the free radicals. Conventionally, the experiments to identify the antioxidant proteins are conducted using biochemical techniques, which however, is arduous and comes at high cost. The protein sequence database provided an opportunity for computer-based antioxidant protein inference [5]. In early study [6], a naive bayes method was developed for the prediction of antioxidant proteins. Amino acid compositions and di-petide compositions of the protein sequences were utilized as features, which were later subjected to a feature selection algorithm. The resultant features with reduced dimension were then used as the training dataset. Later, AoPred was presented by same authors [7], in which g-peptide composition was adopted to extract features from the protein sequences and analysis of variance (ANOVA) [8], was employed to obtain the optimal feature set to train a support vector machines (SVM).

Recently, deep learning (DL) based classification methods have been proposed to perform prediction in almost every field. In particular, many bioinformatics applications have taken advantage of the outstanding capabilities of deep neural networks (DNN) [9]. The prediction of protein structure and function, based on the deep learning algorithms have also been proposed, which yielded highly accurate results [10,11], confirming the potential scope of DL methods in this field. Motivated by the recent success of deep latent-space based learning methods [12,13,14], herein, we propose a deep learning based classification model which is essentially an auto-encoder with an embedded classifier.

The major advantages of the proposed approach is its ability to learn the non-linear embedding of the features whilst maintaining the properties of the original features through reconstruction. This consistency helps filter out fluctuating data points by evaluating their distance from the class centers. As a result, the model can be an important tool for designing a classifier with noisy annotations. Another advantage of using such configuration is the elimination of the need of a crucial step of feature engineering, which involves the selection of dominant features for the training of classifier. The built model has been evaluated by the widely known statistical parameters, and the performance is compared with the existing methods. The results show that the proposed method offers superior performance on the benchmark dataset.

The rest of the paper is organized as follows: In Section 2, the details of the benchmark dataset, features and the classification model are discussed. Section 3 presents the results of the performance evaluation of the proposed method and its comparison with contemporary approaches. The conclusion of the paper is presented in Section 5.

## 2. Materials and Methods

### 2.1. Evaluation Parameters

To evaluate the performance of the proposed method, we used metrics for the imbalanced class samples including sensitivity (Sn) or recall, specificity (Sp), precision, conventional accuracy (ACC), mathews correlation coefficient (MCC), balanced accuracy (BACC), youden’s index (YI), F1 score, and Cohen’s kappa (κ). These parameters can be evaluated using the following equations:

(1) $$Sensitivity=\frac{TP}{TP+FN}$$

(2) $$Specificity=\frac{TN}{TN+FP}$$

(3) $$Precision=\frac{TP}{TP+FP}$$

(4) $$Accuracy=\frac{TP+TN}{TP+TN+FP+FN}$$

(5) $$MCC=\frac{\left(TP\times TN)-(FP\times FN\right)}{\sqrt{\left(TP+FP)\times (TN+FN)\times (TP+FN)\times (TN+FP\right)}}$$

(6) $$Balanced\;Accuracy=\frac{Sensitivity+Specificity}{2}$$

(7) $$Youden^{\prime }s\;Index=Sensitivity+Specificity-1$$

(8) $$F1\;Score=2\times \frac{Precision\times Recall}{Precision+Recall}$$

(9) $$Po=\frac{TP+TN}{TP+TN+FP+FN}$$

(10) $$Pe=\frac{\left((TP+FN)\times (TP+FP)+(FP+TN)\times (FN+TN)\right)}{{\left(TP+TN+FP+FN\right)}^{2}}$$

(11) $$\kappa =\frac{Po-Pe}{1-Pe}$$

Here TP, FP, TN, and FN represent true positive (correctly classified AoPs), false positive (incorrect classification of non-AoP as AoP), true negative (correctly classified non-AoPs), and false negative (incorrect classification of AoP as non-AoP), respectively.

### 2.2. Dataset

The benchmark dataset has been obtained from Feng et al. [6]. The formation of the dataset included the selection of the sequences from UniProtKB, that were annotated and reviewed as antioxidant in the molecular function of gene ontology. The sequences were manually observed to remove any nonstandard letters other than the 20 standard amino acid alphabets. Furthermore, the sequence similarity was reduced to ≤60% using CD-HIT. The final dataset contained 253 antioxidant protein sequences and 1552 non-antioxidant proteins. To fairly compare the performance of the proposed method, the training and test datasets were set quantitatively equal to the contemporary approaches, i.e., 200 antioxidant and 1240 non-antioxidant proteins were used as the training subset, while the remaining 53 antioxidants and 312 non-antioxidant proteins were used as the test dataset.

### 2.3. Features and Latent Space Encoding

**Figure 1:**
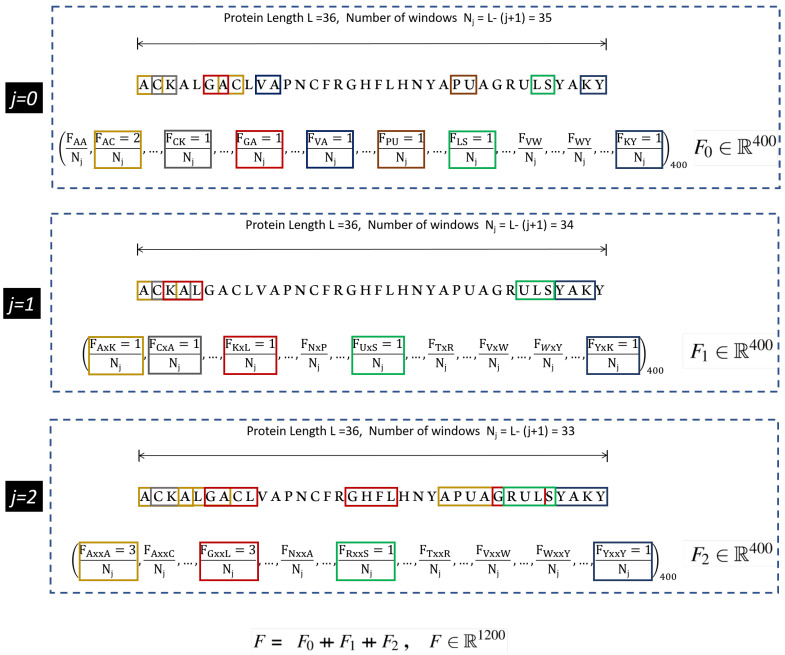
Illustration of CKSAAP descriptor calculation for k=2. Extracted from [13].

The protein features are made compatible with the machine learning algorithms by encoding them numerically. Several encoding schemes have been utilized by the researchers in accordance with the adopted machine learning method [13,15,16,17]. In this study, we use a well known feature encoding method called the composition of *k*-spaced amino acid pairs (CKSAAP). It is a comprehensive encoding scheme, and has shown significant performance in variety of protein prediction tasks [18,19,20]. In CKSAAP, *k* denotes the gap or space between the pairs of the amino acid fragments. For k=2, the intermediate feature vectors ranging from j=0,…,k are obtained, which are stacked together to form the final feature vector. An illustration of the feature vector for k=2, obtained from the CKSAAP method is shown in Figure 1.

As observed in Figure 1, the dimensions of the resultant feature vector of CKSAAP can be very high. This high resolution feature vector has multilevel granularity which intensifies the relationship of amino acid fragments. However, the inadequate number of positive samples leads to the large p small n problem and therefore, feature engineering such as component analysis [21] and information gain [15] must be employed. The feature engineering tends to work well in the circumstances when the features and class labels have a linear relationship, however, in deep learning applications, this relationship is mostly non-linear, which presents complications in selecting the efficacious features. Therefore, in this study we employ an auto-encoder, which is commonly used for the compressed representation of the input data [22]. The compressed input exists in the bottle-neck of the auto-encoder called the latent space. The latent space contains sufficient information to reconstruct the approximate of the original input by the decoder unit. In the proposed method, the latent space also serves as an input to the classifier module, which is a fully connected neural network, the details of which is presented in Section 2.4.

### 2.4. Neural Network Architecture

**Figure 2:**
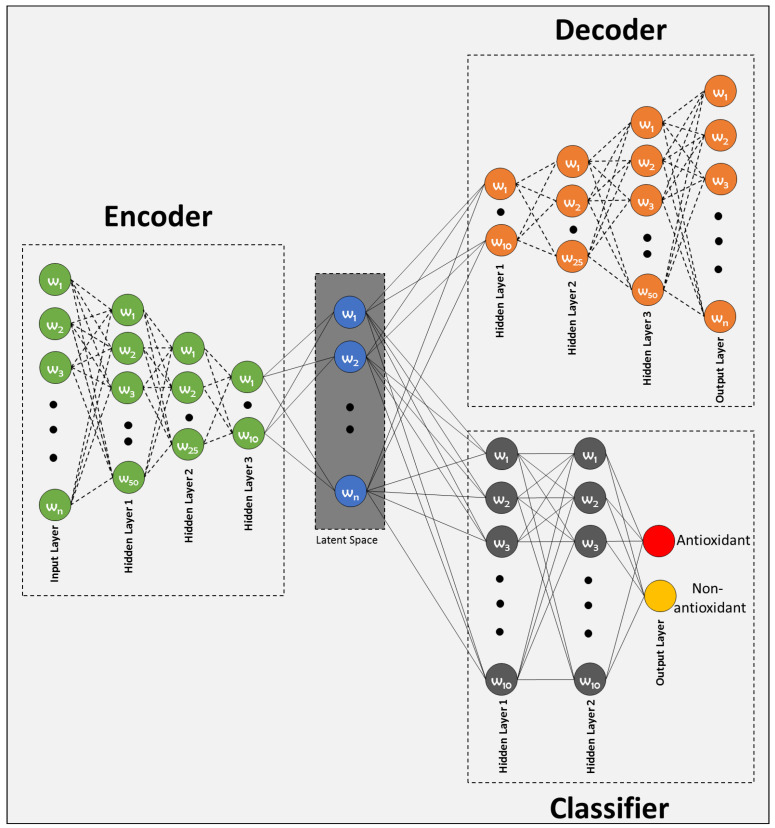
Proposed latent-space encoding-based Antioxidant protein prediction model.

The proposed Deep Latent Space Encoding (DeepLSE) model comprises of three modules: (1) encoder, (2) decoder and (3) classifier. The input layer of the encoder of the baseline model consists of 400×(k+1) neurons, the first and second hidden layers has N×10, N×5 neurons respectively, while the final hidden layer has N×2 neurons. To improve generalization and to avoid over-fitting, the batch-normalization layer and a dropout of 30% has been employed between each dense layer. The decoder being the complement of the encoder and has mirror symmetric configuration. For classification, a multi-layer perceptron has been implemented with two fully connected hidden layers. Each hidden layer has 10 neurons while the output layer has 2 neurons characterizing the antioxidant and non-antioxidant proteins class labels. For N=5, the architecture of the proposed model is shown in Figure 2.

### 2.5. Training Configurations

The model is trained using Python on Tensorflow-Keras [23] platform for different configurations of latent variables (LV) and gap values (*k*). The output of encoder/decoder and classification network uses sigmoid and soft-max activation functions respectively, while the hidden layers of all modules use rectified linear unit (ReLU) activation function to avoid the occurrence of vanishing gradient. Two loss functions; mean squared error and binary cross-entropy for auto-encoder and classifier respectively are being minimized using the default learning rate of *RMSprop optimizer* for 1000 epochs with an early stopping tolerance of 100 epochs. The convex combination of two losses is achieved by

$$L_{combined}=\lambda L_{decoder}+\left(1-\lambda \right)L_{classification},$$

where λ is a mixing weight and which was set to be 0.99.

## 3. Results and Discussion

### 3.1. Ablation Study

#### 3.1.1. Finding Best Latent-Space Encoding (LSE) Scheme

**Figure 3:**
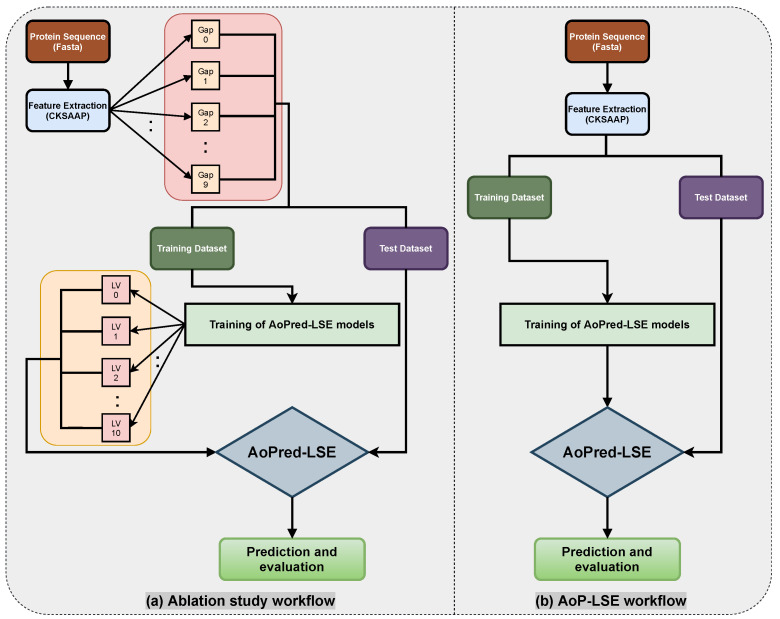
Workflow and ablation study diagram for AoP-LSE method.

The workflow of the proposed study is aimed to obtain the best classification model based on two variable parameters, i.e., the gap between the two amino acid pairs, which is done by setting different values of *k* during the encoding, and the number of units in the latent space *LS*. An ablation study has been designed to acquire latent-space encoding (LSE) with varying number of aforementioned variable parameters according to the workflow depicted in Figure 3a. The proteins are distributed into train and test datasets as discussed in the Section 2.2 and resultant datasets are processed for CKSAAP encoding. For the model configuration (N=5) shown in Figure 2, nine different subsets are constructed by keeping k=1 to 9. For each value of *k*, the model is trained with several values of latent space variables LVs ranging from 2 to 10. For each configuration, 20 independent trials are performed and the obtained test results are averaged. Same procedure is followed for the next configuration by incrementing the gap value (*k*) between the amino acid pairs. The model with the best average results is finally selected as the base model to perform prediction and is named as AoP-LSE.

**Figure 4:**
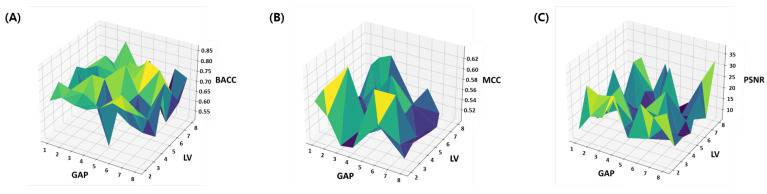
Ablation study: Number of latent variables (LV) and Gaps (*k*) in CKSAAP features are optimized within 4×9 parameter space. (**A**) Balanced Accuracy, (**B**) MCC and (**C**) PSNR.

The summary of the ablation study is provided in a visual and tabular form. The results of the BACC, MCC and PSNR are provided as a surface plot in Figure 4. These metrics are suggested to be an effective evaluation parameter for the imbalanced classification problems. From the results it can be seen that for the large range of parameters, the model achieve comparable accuracy and the variation in both the BACC and MCC is under 10%. However the PSNR performance is highly sensitive to parameter choice as the variation in PSNR around 20 dB units. This clearly indicates discrepancy in most descriptive and most discriminating features. In other words, embedded features which are useful for the reconstruction of original feature space are not necessarily the most discriminating features for designing the classifier, while the most discriminating embedded features are not necessarily the most descriptive (indicative feature for oxidation properties) features. Therefore, finding a balanced features embedding that can help classification while preserving the indicative feature for oxidation properties is important. The same issue is further discussed in the Section 4.1 where learned latent-spaces are compared.

Table 1 presents the results of the balanced accuracy, which is the average of recall of each class and is suggested to be an effective evaluation parameter for the imbalanced classification problems. The consistent values of standard deviations and mean for 20 random trials manifests the stability of the model. However, the best average balanced accuracy results (i.e., 76.2%) was obtained when the value of both *k* and LV was equal to 6. Therefore, the model with k=6 and LV=6 has been selected for further testing and evaluations.

#### 3.1.2. Finding Best-Configuration for DeepLSE Architecture

For the aforementioned combination of k=6, and LV=6, three configurations of model parameters were evaluated. The number of neurons in each configuration were incremented as shown in Table 2, and their effect on the classification performance were evaluated for the metrics including Youden’s index, MCC, receiver operating characteristics area under the curve (ROC AUC), precision recall area under the curve (PR AUC) and the mean reconstruction error. 10 independent trials were performed for each configuration and the one producing the best results in terms of aforementioned performance metrics was selected as the baseline model in this study. Table 3 shows the results of the ablation study.

It was observed from the analysis above that a shallow model (N=1) failed to learn the discriminating features, whereas a highly complex model (N=10) also showed mediocre performance. Interestingly, the moderate size model with the configuration (N=5) works best. Although, this ablation study is not exhaustive, nevertheless, it gives sufficient clue that the selected parameters are the optimal choice for best performance.

### 3.2. Comparison with the Contemporary Methods

Once the best model has been identified from the ablation study, it is selected for further testing and evaluation according to the workflow depicted in Figure 3b. For comparative analysis, the model is compared with the Naive Bayes [6] and AODPred(SVM) [7]. Table 4 presents a comparison of the evaluation metrics of the proposed AoP-LSE including the test dataset Accurary (ACC), Sensitivity ($S_{n}$), Specificity ($S_{p}$), and Youden’s Index (YI) with Naive Bayes [6] and AODPred(SVM) [7]. Although the sensitivity of proposed method is relatively lower, the proposed AoP-LSE method achieved 0.14, and 0.03 units higher Youden’s index value than the Naive Bayes and AODPred(SVM) methods respectively. Since, for a highly skewed test dataset, the higher values of a balanced metric e.g., Youden’s index are much desired than the individual class metric, therefore, the proposed AoP-LSE can be considered as a better classification approach.

### 3.3. Verification on Independent-Dataset of Antioxidant Proteins

For objective evaluation of the performance of the proposed method, we utilize the reviewed antioxidant proteins from UniProtKB/Swiss-Prot [24]. UniProtKB is a high quality, manually annotated and non-redundant protein sequence database, which brings together experimental results, computed features and scientific conclusions. These sequences were totally independent and were not present in the positive of negative datasets of the proposed study. Comparison was performed among AODPred [7], Vote9 [25] and the proposed AoP-LSE methods. The results are presented in Table 5 show that the proposed method successfully predicts 21 out of 22 independent antioxidant proteins achieving an accuracy of 95.4%. This superior performance of of AoP-LSE indicates that it can be utilized as a useful tool for the annotation of unknown antioxdants.

## 4. Analysis of Deep Latent-Space Encoding

### 4.1. Comparison of Feature and Latent-Space Discrimination Capability

**Figure 5:**
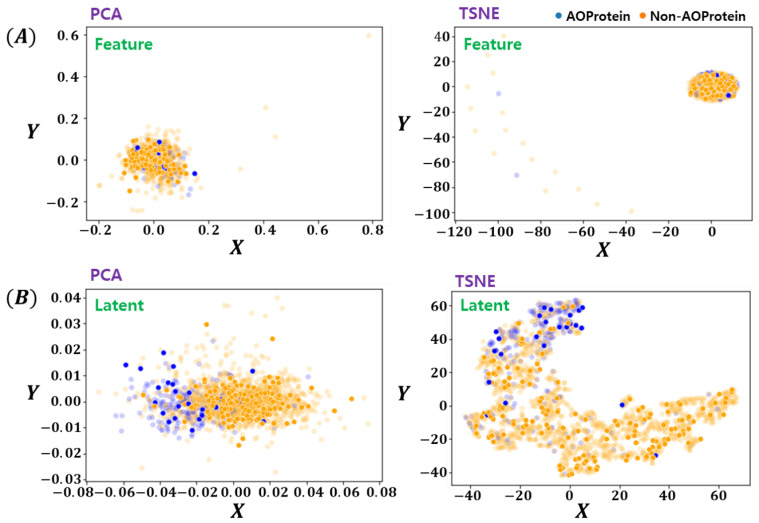
Visualization of Feature and Latent Space Embedding by PCA & tSNE. (**A**) Feature Space Embeddings (PCA, tSNE), and (**B**) Latent Space Embeddings (PCA, tSNE). The opaque points are the training instances and clearly expressed points are test instances.

The latent space learned by the proposed method has been visualized in Figure 5. For fair comparison, two feature-encoding/ dimension-reduction methods are utilized. For linear and non-linear embedding, PCA [26] and tSNE [27] methods are used respectively. Both methods were provided with the original feature space and deep encoded latent-space data. From Figure 5 it can be clearly seen that the proposed deep latent space encoding method learns to separate the two classes data which are not distinct in the original feature space as can be seen in Figure 5A. The tSNE results in Figure 5B shows interesting patterns, indicating the possibility of noisy labels, which are not as prominent in original features space. Another important characteristics of proposed method is the fact that even with linear encoding method of PCA, the proposed Deep-LSE encoded features looks linearly separable, which is an added advantage as in many bioinformatics problem the simplicity and explainability is more important than the gain in classification scores.

Since both classification and reconstruction modules are trained simultaneously and the training attention is increased towards minimizing the reconstruction loss. The model has more freedom to represent individual samples with its variability. On the other hand, the attention of learning is towards minimizing the decoder’s loss with a low value of the classification weight. It creates an unfair tug of war between the two objectives and results in a cluster of high dimensional data in a compact distinguishable latent-space.

### 4.2. Comparison of Proposed DeepLSE and Conventional Auto-Encoder-Based Encoding Schemes

Although, the architecture of the proposed model is similar to auto-encoder (AE) + multi-layer perceptron (MLP) neural network. However, the way it has been configured/trained i.e., combined training of AE+MLP makes it different from the conventional approach where AE is trained separately for dimension reduction and obtain the most representative (low-ranked) features in the latent space (LS) for signal reconstruction. The proposed DeepLSE on the other hand, not only learns the most representative features but also impose a classification constraint on the latent variables. Thus, creating a tug of war between the decoder and the classifier forcing the encoder to generate useful features. To emphasize this point and for a fair comparison with existing techniques, an evaluation on three different models has been presented.

We trained the auto-encoder network with similar configuration (N=5, k=6 and LV=6) as in DeepLSE and extracted the latent space encoding for the entire dataset. Later the encoder latent-variables were used for the design and evaluation of auto-encoder+multi layer perceptron (AE + MLP), auto-encoder+ support vector machine (AE + SVM) and auto-encoder+naive bayes (AE + NB) methods. The models were evaluated for the several evaluation parameters and the results are reported in Table 6. For AE + MLP model, the same MLP configuration was used as in DeepLSE. For AE+SVM, linear SVM [28,29] with Euclidean-distance-based radial-basis-function [30] kernel, balanced class weights and l2 penalty of C=1000 is used. For AE + NB method, Gaussian Naive Bayes [31] with prior of (0.5, and 0.5) is used. Both the SVM and NB methods were implemented using *scikit-learn* package [32] on Python 3.

**Figure 6:**
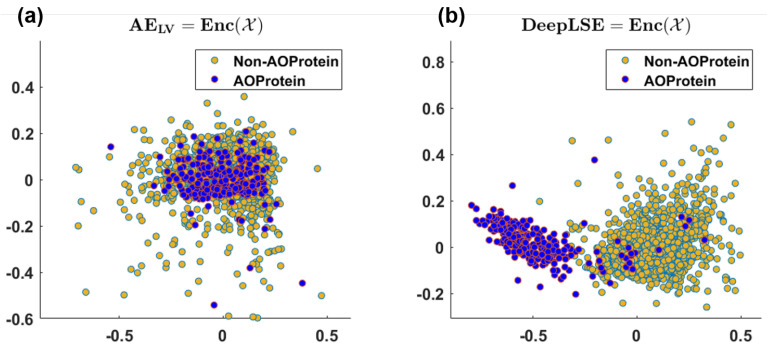
2D visualization of (**a**) latent-space embedding of conventional auto-encoder, and (**b**) latent-space embedding of proposed DeepLSE-based Auto-Encoder neural network using PCA. (**a**) Training/Test MSE (dB): 46.21/46.37, and 1D-PCA-GCNR score 0.22, (**b**) Training/Test MSE (dB): 45.05/44.88 and 1D-PCA-GCNR score 0.91.

As anticipated, the conventional AE fail to generate useful embedding, hence, the performance of all the aforementioned models is poor. To further highlight this point, we provided the visualization for the AE and DeepLSE latent variables in Figure 6, and compare the discrimination potentiality of the original feature-space of DeepLSE’s and AE’s latent-variables using one-dimensional PCA with absolute-GSSMD [33] method. The GSSMD is derived from generalized contrast-to-noise ratio (GCNR) [34] metric, in which the overlap between two distributions is compared. For an ideal classification, a GCNR score of 1 is obtained, which suggests that the two distributions are distinct with no overlap. While for classification in worst-case, the two distribution must be fully overlapping resulting in the GCNR score of 0. It can be observed in Figure 6a, that the conventional AE, with only dimension reduction constraint, obtained the training and testing MSE of 46.21 dB and 46.37 dB, respectively with (1D-PCA-GCNR score = 0.22) and does not distinguishes the two classes. While with a comparable MSE error, the proposed method depicted in Figure 6b, presents superior learning capabilities (1D-PCA-GCNR score = 0.91) and maps the AoP and non-AoP in separate regions.

### 4.3. Analysis of Decoder and Residual Error

**Figure 7:**
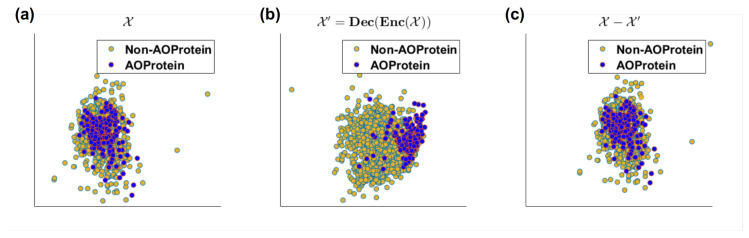
2D visualization of (**a**) original feature space (1D-PCA-GCNR score 0.14), (**b**) decoded-output using proposed DeepLSE(1D-PCA-GCNR score 0.77) and (**c**) residual of original-input and decoded output of DeepLSE neural network using PCA (1D-PCA-GCNR score 0.12).

Herein, we analyzed the discriminating power of input, decoded and residual signals. This provide another interesting characteristics of our proposed DeepLSE. From the 2D visualization of original feature space X, the decoded-output using proposed DeepLSE $X^{\prime }=\mathrm{Dec}\left(\mathrm{Enc}\left(X\right)\right)$, and the residual of original-input and decoded output of DeepLSE $X-X^{\prime }$ in Figure 7a it can be seen that the original feature space X with 1D-PCA-GCNR score of 0.14 units, is highly overlapping/non-linear and have almost no discriminating capabilities in linear-space. Whereas, the proposed DeepLSE method which acts as optimal-transport (OT) [35,36], and shift the data distributions in such a way that the reconstruction error of the original signal remains comparable to the conventional AE as well as the classification power is greatly improved. Its feature space, shown in Figure 7b, results in 1D-PCA-GCNR score of 0.77 units, which is 0.63 units higher than the original discriminating power. The 2D projection of residual error signal $X-X^{\prime }$ in Figure 7c looks identical to the original feature space X, however, the class separability in residual error is dropped by 0.02 units, This is understandable as the ideal residual error should not contain any useful information which may improve the classification.

## 5. Conclusions

In this study, we proposed a deep latent space encoding method for the classification of anti-oxidant proteins using sequence derived features. In particular, the composition of k-spaced amino acid pair (CKSAAP) and a densely connected multi-layered perception neural network are used which are trained in tandem. The proposed method can be used to extract a non-redundant compact feature space, which is shown to outperform the conventional antioxidation protein classification approaches. Furthermore, the effect of varying parameters and number of gaps in CKSAAP and latent variables in Deep-LSE is analyzed, which suggests that a sufficient separable encoding can be learned by keeping moderate number of neurons in the architecture with k=6 and LV=6. The proposed approach effectively learns the non-linear embedding of the features from original feature space, thus filters out the non-relevant data on the basis of their distance from the class centers. This property leads towards the development of a classifier for the antioxidant proteins with noisy annotations and for assessment, the proposed method has been evaluated on an independent dataset which showed superior classification performance compared to the contemporary methods. We hope that AoP-LSE will serve as an effective method for the identification of unknown antioxidants.

## Figures and Tables

**Table 1:** Balanced accuracy results of ablation study on Gap(*k*), and LV parameters.

Gap/*LV*	2	3	4	5	6	7	8	9
k=1	0.739±0.082	0.713±0.070	0.716±0.091	0.688±0.094	0.710±0.047	0.717±0.139	0.726±0.049	0.704±0.109
k=2	0.701±0.080	0.725±0.068	0.715±0.086	0.675±0.079	0.717±0.057	0.748±0.026	0.641±0.124	0.654±0.093
k=3	0.729±0.083	0.759±0.034	0.702±0.110	0.716±0.061	0.668±0.124	0.703±0.083	0.722±0.094	0.669±0.117
k=4	0.722±0.080	0.648±0.107	0.702±0.082	0.708±0.089	0.685±0.111	0.670±0.108	0.678±0.116	0.752±0.051
k=5	0.721±0.036	0.670±0.120	0.688±0.105	0.707±0.096	0.680±0.090	0.708±0.078	0.703±0.073	0.702±0.064
k=6	0.689±0.114	0.702±0.087	0.692±0.085	0.717±0.077	0.762±0.062	0.739±0.061	0.740±0.096	0.753±0.019
k=7	0.699±0.078	0.721±0.079	0.678±0.066	0.713±0.044	0.703±0.098	0.713±0.075	0.657±0.095	0.710±0.098
k=8	0.720±0.062	0.711±0.069	0.636±0.128	0.694±0.104	0.676±0.101	0.681±0.106	0.725±0.034	0.703±0.098
k=9	0.718±0.065	0.708±0.086	0.689±0.066	0.729±0.085	0.694±0.098	0.734±0.063	0.694±0.120	0.720±0.045

**Table 2:** Number of neurons in hidden layers of the architecture for the tested configurations.

Configuration (*N*)	1	5	10
Encode	10-5-2	50-25-10	100-50-20
Decode	2-5-10	10-25-50	20-50-100
Classifier	2-2	10-10	20-20

**Table 3:** Training and test results of ablation study performed on network parameters.

Metric/Configuration (*N*)	1		5		10	
	Train	Test	Train	Test	Train	Test
Youden’s Index	0.53±0.31	0.35±0.20	0.95±0.08	0.59±0.06	0.86±0.08	0.52±0.07
MCC	0.35±0.20	0.45±0.31	0.83±0.15	0.47±0.06	0.73±0.14	0.42±0.06
ROC-AUC	0.80±0.16	0.70±0.12	0.99±0.01	0.84±0.03	0.97±0.02	0.81±0.03
PR-AUC	0.60±0.21	0.46±0.12	0.95±0.05	0.60±0.07	0.84±0.14	0.52±0.11
MSE (dB)	40.81±6.63	42.90±5.27	39.92±4.49	39.82±4.65	35.13±4.47	36.70±4.73

**Table 4:** Performance statistics of (k=6, LV=6) model.

Method	Accuracy	$S_{n}$	$S_{p}$	Precision	YI	BACC	MCC	F1	κ
Naive Bayes [6]	0.668	0.720	0.660	0.26	0.38	0.690	0.27	0.38	0.22
AODPred(SVM) [7]	0.747	0.750	0.744	0.33	0.49	0.747	0.36	0.46	0.32
AoP-LSE (DL)	0.824	0.674	0.849	0.43	0.52	0.762	0.43	0.52	0.42

**Table 5:** Prediction results for 22 independent antioxidant proteins. “✓” indicates correct identification while “✗” represents a incorrect identification.

UniProtKB ACC	NCBI Definition	AODPred	Vote9	AoP-LSE
P9WQB7	Alkyl hydroperoxide reductase C	✓	✗	✓
P9WHH9	Dihydrolipoyl dehydrogenase	✗	✗	✓
P9WIS7	Dihydrolipoyllysine-residue	✗	✓	✓
P9WG35	Thiol peroxidase	✓	✗	✓
P9WGE9	Superoxide dismutase	✓	✗	✓
P9WQB5	Alkyl hydroperoxide reductase	✓	✗	✓
P9WIE3	Alkyl hydroperoxide reductase	✓	✗	✓
P0CU34	Peroxiredoxin TSA1	✓	✗	✓
Q5ACV9	Cell surface superoxide dismutase	✗	✗	✓
P9WHH8	Dihydrolipoyl dehydrogenase	✗	✓	✓
P9WIE1	Putative peroxiredoxin Rv2521	✗	✓	✓
P9WIS6	Dihydrolipoyllysine-residue	✗	✗	✓
P9WQB6	Alkyl hydroperoxide reductase	✓	✗	✓
P9WID9	Putative peroxiredoxin Rv1608c	✓	✗	✓
O17433	Cys peroxiredoxin	✓	✗	✗
P9WIE0	Putative peroxiredoxin MT2597	✗	✗	✓
P9WID8	Putative peroxiredoxin MT1643	✓	✗	✓
P9WGE8	Superoxide dismutase [Cu-Zn]	✓	✗	✓
C0HK70	Superoxide dismutase	✓	✗	✓
P9WQB4	Alkyl hydroperoxide reductase AhpD	✓	✗	✓
P9WG34	Thiol peroxidase	✓	✗	✓
P9WIE2	Alkyl hydroperoxide reductase E	✓	✗	✓

**Table 6:** Evaluation of different models trained on the latent variables of an auto-encoder on complete dataset.

Method	Sensitivity	Specificity	Accuracy	BACC	MCC	F1 Score	YI
AE + MLP	0.65	0.57	0.58	0.61	0.16	0.31	0.23
AE + SVM	0.64	0.56	0.57	0.50	0.15	0.30	0.21
AE + NB	0.78	0.36	0.42	0.57	0.11	0.28	0.15
Proposed DeepLSE	0.67	0.84	0.82	0.76	0.43	0.52	0.52

## References

[1] Chauvin J.P.R., Griesser M., Pratt D.A. (2019). The antioxidant activity of polysulfides: It’s radical!. Chem. Sci..

[2] Sannasimuthu A., Arockiaraj J. (2019). Intracellular free radical scavenging activity and protective role of mammalian cells by antioxidant peptide from thioredoxin disulfide reductase of Arthrospira platensis. J. Funct. Foods.

[3] Tang J., Fu J., Wang Y., Luo Y., Yang Q., Li B., Tu G., Hong J., Cui X., Chen Y. (2019). Simultaneous improvement in the precision, accuracy, and robustness of label-free proteome quantification by optimizing data manipulation chains. Mol. Cell. Proteom..

[4] Grzesik M., Bartosz G., Stefaniuk I., Pichla M., Namieśnik J., Sadowska-Bartosz I. (2019). Dietary antioxidants as a source of hydrogen peroxide. Food Chem..

[5] Feng P., Ding H., Lin H., Chen W. (2017). AOD: The antioxidant protein database. Sci. Rep..

[6] Feng P.M., Lin H., Chen W. (2013). Identification of antioxidants from sequence information using naive Bayes. Comput. Math. Methods Med..

[7] Feng P., Chen W., Lin H. (2016). Identifying antioxidant proteins by using optimal dipeptide compositions. Interdiscip. Sci. Comput. Life Sci..

[8] St L., Wold S. (1989). Analysis of variance (ANOVA). Chemom. Intell. Lab. Syst..

[9] Li H., Tian S., Li Y., Fang Q., Tan R., Pan Y., Huang C., Xu Y., Gao X. (2020). Modern deep learning in bioinformatics. J. Mol. Cell Biol..

[10] Senior A.W., Evans R., Jumper J., Kirkpatrick J., Sifre L., Green T., Qin C., Žídek A., Nelson A.W., Bridgland A. (2020). Improved protein structure prediction using potentials from deep learning. Nature.

[11] Torrisi M., Pollastri G., Le Q. (2020). Deep learning methods in protein structure prediction. Comput. Struct. Biotechnol. J..

[12] Park S., Khan S., Wahab A. (2020). E3-targetPred: Prediction of E3-Target Proteins Using Deep Latent Space Encoding. arXiv.

[13] Usman M., Khan S., Lee J.A. (2020). Afp-LSe: Antifreeze proteins prediction Using Latent Space encoding of composition of k-Spaced Amino Acid pairs. Sci. Rep..

[14] Al-Saggaf U.M., Usman M., Naseem I., Moinuddin M., Jiman A.A., Alsaggaf M.U., Alshoubaki H.K., Khan S. (2021). ECM-LSE: Prediction of Extracellular Matrix Proteins using Deep Latent Space Encoding of k-Spaced Amino Acid Pairs. Front. Bioeng. Biotechnol..

[15] Khan S., Naseem I., Togneri R., Bennamoun M. (2016). Rafp-pred: Robust prediction of antifreeze proteins using localized analysis of n-peptide compositions. IEEE/ACM Trans. Comput. Biol. Bioinform..

[16] Naseem I., Khan S., Togneri R., Bennamoun M. (2017). ECMSRC: A sparse learning approach for the prediction of extracellular matrix proteins. Curr. Bioinform..

[17] Usman M., Khan S., Park S., Wahab A. (2021). AFP-SRC: Identification of Antifreeze Proteins Using Sparse Representation Classifier. Neural Comput. Appl..

[18] Mosharaf M.P., Hassan M.M., Ahmed F.F., Khatun M.S., Moni M.A., Mollah M.N.H. (2020). Computational prediction of protein ubiquitination sites mapping on Arabidopsis thaliana. Comput. Biol. Chem..

[19] Usman M., Lee J.A. Afp-cksaap: Prediction of antifreeze proteins using composition of k-spaced amino acid pairs with deep neural network. Proceedings of the 2019 IEEE 19th International Conference on Bioinformatics and Bioengineering (BIBE).

[20] Ju Z., Wang S.Y. (2020). Prediction of lysine formylation sites using the composition of k-spaced amino acid pairs via Chou’s 5-steps rule and general pseudo components. Genomics.

[21] Zhao H., Zheng J., Xu J., Deng W. (2019). Fault diagnosis method based on principal component analysis and broad learning system. IEEE Access.

[22] Yoon Y.H., Khan S., Huh J., Ye J.C. (2018). Efficient b-mode ultrasound image reconstruction from sub-sampled rf data using deep learning. IEEE Trans. Med. Imaging.

[23] Chollet F. (2015). Keras. https://keras.io.

[24] Consortium U. (2019). UniProt: A worldwide hub of protein knowledge. Nucleic Acids Res..

[25] Li X., Tang Q., Tang H., Chen W. (2020). Identifying antioxidant proteins by combining multiple methods. Front. Bioeng. Biotechnol..

[26] Jolliffe I.T. (1986). Principal components in regression analysis. Principal Component Analysis.

[27] Van der Maaten L., Hinton G. (2008). Visualizing data using t-SNE. J. Mach. Learn. Res..

[28] Chang C.C., Lin C.J. (2011). LIBSVM: A library for support vector machines. ACM Trans. Intell. Syst. Technol. (TIST).

[29] Fan R.E., Chang K.W., Hsieh C.J., Wang X.R., Lin C.J. (2008). LIBLINEAR: A library for large linear classification. J. Mach. Learn. Res..

[30] Khan S., Naseem I., Togneri R., Bennamoun M. (2017). A novel adaptive kernel for the rbf neural networks. Circuits Syst. Signal Process..

[31] Rennie J.D., Shih L., Teevan J., Karger D.R. Tackling the poor assumptions of naive bayes text classifiers. Proceedings of the 20th International Conference on Machine Learning (ICML-03).

[32] Pedregosa F., Varoquaux G., Gramfort A., Michel V., Thirion B., Grisel O., Blondel M., Prettenhofer P., Weiss R., Dubourg V. (2011). Scikit-learn: Machine Learning in Python. J. Mach. Learn. Res..

[33] Park S., Khan S., Moinuddin M., Al-Saggaf U.M. GSSMD: A new standardized effect size measure to improve robustness and interpretability in biological applications. Proceedings of the 2020 IEEE International Conference on Bioinformatics and Biomedicine (BIBM).

[34] Rodriguez-Molares A., Rindal O.M.H., D’hooge J., Måsøy S.E., Austeng A., Bell M.A.L., Torp H. (2019). The generalized contrast-to-noise ratio: A formal definition for lesion detectability. IEEE Trans. Ultrason. Ferroelectr. Freq. Control.

[35] Peyré G., Cuturi M. (2019). Computational optimal transport: With applications to data science. Found. Trends Mach. Learn..

[36] Khan S., Huh J., Ye J.C. (2021). Variational Formulation of Unsupervised Deep Learning for Ultrasound Image Artifact Removal. IEEE Trans. Ultrason. Ferroelectr. Freq. Control.

